# Review on the Application of Hyperspectral Imaging Technology of the Exposed Cortex in Cerebral Surgery

**DOI:** 10.3389/fbioe.2022.906728

**Published:** 2022-05-27

**Authors:** Yue Wu, Zhongyuan Xu, Wenjian Yang, Zhiqiang Ning, Hao Dong

**Affiliations:** ^1^ Research Center for Intelligent Sensing Systems, Zhejiang Lab, Hangzhou, China; ^2^ Anhui Institute of Optics and Fine Mechanics, Chinese Academy of Sciences (CAS), Hefei, China; ^3^ Science Island Branch, Graduate School of USTC, Hefei, China; ^4^ Research Center for Sensing Materials and Devices, Zhejiang Lab, Hangzhou, China

**Keywords:** hyperspectral imaging, biomedical, cerebral disease, brain tissue metabolic and hemodynamic, brain cancer diagnosis

## Abstract

The study of brain science is vital to human health. The application of hyperspectral imaging in biomedical fields has grown dramatically in recent years due to their unique optical imaging method and multidimensional information acquisition. Hyperspectral imaging technology can acquire two-dimensional spatial information and one-dimensional spectral information of biological samples simultaneously, covering the ultraviolet, visible and infrared spectral ranges with high spectral resolution, which can provide diagnostic information about the physiological, morphological and biochemical components of tissues and organs. This technology also presents finer spectral features for brain imaging studies, and further provides more auxiliary information for cerebral disease research. This paper reviews the recent advance of hyperspectral imaging in cerebral diagnosis. Firstly, the experimental setup, image acquisition and pre-processing, and analysis methods of hyperspectral technology were introduced. Secondly, the latest research progress and applications of hyperspectral imaging in brain tissue metabolism, hemodynamics, and brain cancer diagnosis in recent years were summarized briefly. Finally, the limitations of the application of hyperspectral imaging in cerebral disease diagnosis field were analyzed, and the future development direction was proposed.

## 1 Introduction

Brain, the most important and complex organ in human body, is the vehicle for our cognition, emotion, mobility, language, memory, consciousness and self-awareness (Salles et al., 2019). Thanks to the invention of non-invasive imaging of the human brain, our understanding of the relationship between the brain and behaviors has undergone a major shift. Nowadays, a variety of imaging modalities are employed as guidance tools during the neurosurgeries e.g., computed tomography (CT) (Paty et al., 1988; Hövels et al., 2008), magnetic resonance imaging (MRI) (Dewenter et al., 2021; Xu et al., 2021) and fluorescent tumor markers (FTM) (Ferraro et al., 2016). Nevertheless, these methods have some limitations. For instance, CT is harmful to the patient’s brain tissue because of the higher radiation (Pelizzari et al., 1989; Gong et al., 2007). MRI has poor spatial resolution, significantly prolongs the operation duration, and only a certain quantity of images are available (Ganser et al., 1997). FTM is able to identify tumor boundaries, but it can only be used for high-grade tumors due to the patient-related chain reaction. These technologies have higher probabilities to cause terrible clinical results due to incomplete excision of diseased tissue or resection of adjacent normal tissue (Torbey et al., 2015). Therefore, a label-free and contactless imaging approach is urgently demanded to assist physicians during neurosurgery (Li et al., 2013; Lu, 2014; Halicek et al., 2019a).

Hyperspectral imaging (HSI), as an optical detection technique, is aimed to record the spectrum of each pixel in an image. In this sense, HSI is a natural extension of color (RGB) imaging. HSI has the advantage of acquiring two-dimensional images over a wide range of the electromagnetic spectrum and has numerous practical applications, including Oceanic exploration (Freitas et al., 2021; Wang, 2021), food quality and safety organizations (Lohumi, 2018; Kim et al., 2022), disaster monitoring (Farhadi et al., 2022), remote sensing (Abdulridha et al., 2019), and agriculture (Lu, 2020).

Recently, as a promising optical technology, HSI is extensively utilized in the field of biomedical engineering, for life science research, non-invasive diagnostics and image-guided surgery (Lu and Fei, 2014; Ortega et al., 2019; Fei and Amigo, 2020). In the past decades, there were two primary factors that aroused the interests of medical researchers for HSI technology. First, the interaction between electromagnetic radiation and tissues contains quantitative diagnostic information on histopathology. Second, for its non-invasive nature, HSI can provide real-time information of several biological processes in healthy and diseased tissues. Specifically, HSI measures the intensity changes at multiple wavelengths, demonstrating the reflection, emission or fluorescence interactions with the target tissues, which indicate the changes in the biological structure of its components and changes in the concentration of intrinsic light-absorbing or luminescent chromophores. Researchers have demonstrated the ability of HSI to detect a wide range of diseases, such as oximetry of the retinal (Gao et al., 2012; Hadoux et al., 2019; Lim et al., 2021), intestinal ischemia identification (Barberio et al., 2020; Mehdorn et al., 2020), histopathological tissue analysis (Khouj et al., 2018), detecting cancer metastases in lung and lymph node tissue (Zhang et al., 2021), blood vessel visualization enhancement (Bjorgan et al., 2015; Fouad Aref et al., 2021), identifying skin tumors (Leon et al., 2020; Courtenay et al., 2021), evaluating the cholesterol levels (Milanic et al., 2015), diabetic foot, etc. In the field of oncology, HSI technology has been successfully applied to detect head and neck cancer (Halicek et al., 2017; Eggert et al., 2022), thyroid and salivary glands (Halicek et al., 2020), gastric cancer (Li et al., 2019; Liu et al., 2020a), oral cancer (Jeyaraj et al., 2020), colon cancer (Baltussen et al., 2019; Manni, 2020; Maktabi, 2021) as well as breast cancer (Kho et al., 2019; Aboughaleb et al., 2020). Previously, other authors have published comprehensive overviews concerning the application of HSI in gastroenterology (Ortega et al., 2019), wound care (Saiko et al., 2020) or breast cancer therapy and diagnosis (Aref et al., 2020). However, to our knowledge, the application of HSI in cerebral disease has not been systematically reviewed.

The purpose of this review is to provide an overview of the main advanced studies concerning the use of hyperspectral imaging technology in cerebral disease diagnosis. The fundamental principles of HSI techniques are presented in detail, and their latest research applications in brain tissue metabolism, hemodynamics and brain cancer diagnosis are summarized and highlighted. In addition, issues encountered in HSI techniques are included and future trends in cerebral disease diagnosis applications are also discussed in the current review. It is noteworthy that this review focus on the application of HSI Technology of the exposed cortex in cerebral surgery. The diffuse HSI Technology in cerebral disease, such as neonatal, aging, neurodegenerative, and cardiac arrest/surgery brain monitoring applications are not included.

## 2 Basic Knowledge of Hyperspectral Image System

HSI uses hundreds of spectral bands, providing more information about the imaging target. The fundamental theory of HSI technology is that all target materials reflect, scatter or absorb energy in different ways due to differences in chemical composition and physical structure when subjected to electromagnetic radiation sources in different wavelength ranges. Light scattering is related to the particle diameter, cell structure, tissue composition and other physical properties of the target materials, HSI while light absorption is concerned with the chemical composition of the target materials. For biomedical applications, HSI can provide an easier way to identify any abnormality in any tissue or organ in the body, allowing for better identification and treatment of disease (Carrasco et al., 2003).

### 2.1 Experimental Devices

The commonly used experimental devices of HSI include light sources, wavelength dispersion modules, and photoelectric detectors. Typically, HSI systems use different detectors to cover different wavelength ranges (Lu, 2014). Charge-coupled devices (CCDs) or complementary metal oxide semiconductors (CMOS) typically encompass the spectral range from 400 to 1000 nm (visible and near-infrared, VNIR), while indium gallium arsenide (InGaAs) and mercury cadmium telluride (HgCdTe) sensors are employed to cover the range of 900–1700 nm (Near Infrared, NIR), 900–2500 nm (Near Short Wave Infrared, SWIR) and 2500–25,000 nm (Middle Infrared, MIR), respectively. Furthermore, HSI systems have a variety of different methods that are differentiated by how the hypercube is generated. They are differentiated according to the type of dispersive element and how the dispersive element projects the acquired information into the detector array. The three typical categories of HSI modes are: Spectral Scan, Spatial Scan and Snapshot HSI systems (Lucas et al., 2004; Gao and Smith, 2015).

**FIGURE 1:**
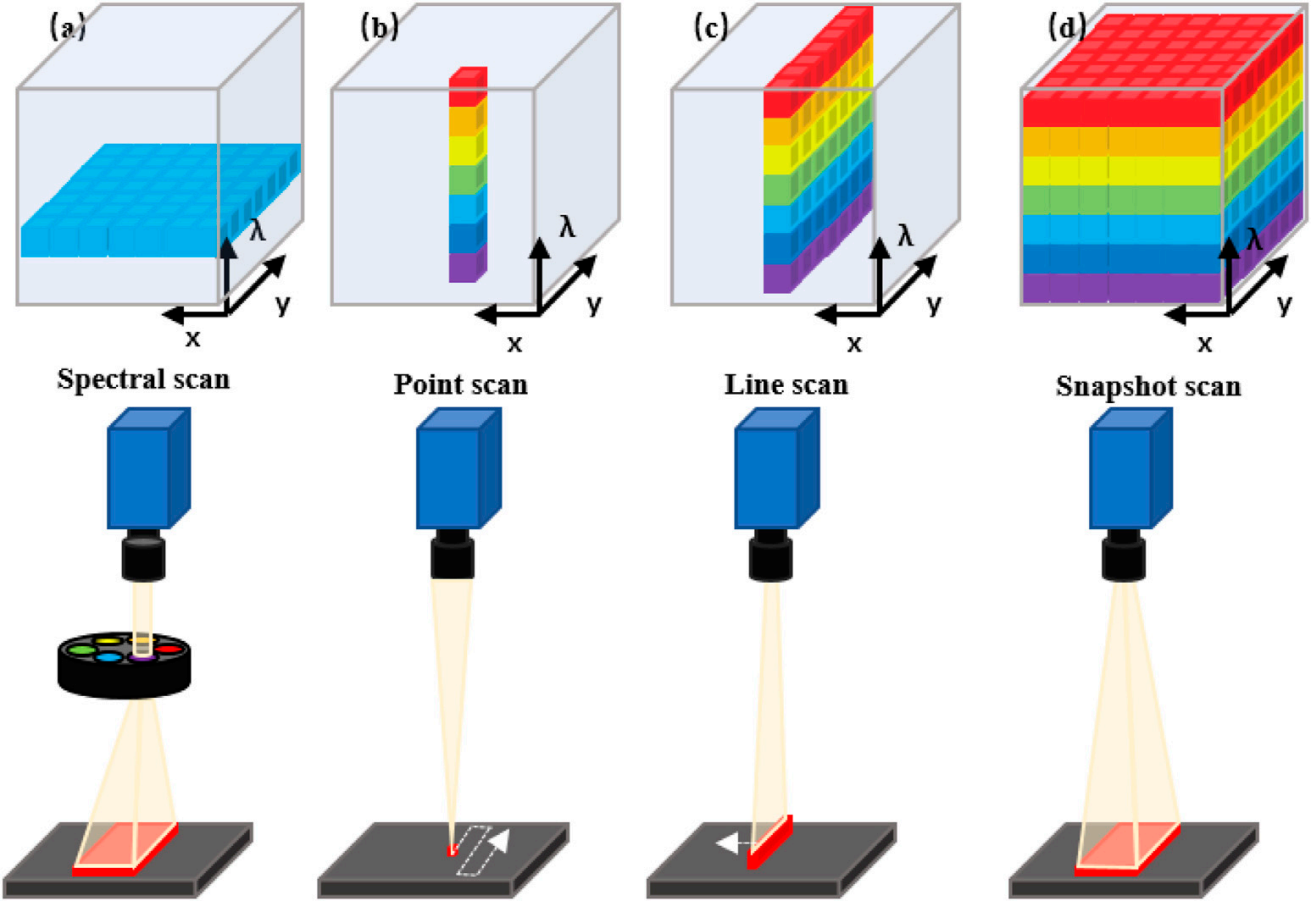
Schematic diagram of the acquisition approach of the hyperspectral data cube. **(A)** Spectral scanning: one wavelength band image at a time. **(B)** Point scanning (wiskbroom imaging): one spectrum of only one point in a single measurement. **(C)** Line scanning (pushbroom imaging): spectra of points on the same line in a single measurement. **(D)** Snapshot: covering the full spectrum in a single measurement.

Spectral scanning HSI systems (also be called as staring HSI systems), just as its name implies, generate hypercubes in one single spectral wavelength (Figure 1A). Basically, each of the obtained images occupies a single spatial slice (x, y) of the hypercube. The hypercube can be reconstructed at each image acquisition by stacking all slices along the spectral dimension (λ). Spatial scanning HSI systems can obtain all spectral information from one spatial scanning of the imaging area in each acquisition process and typically use a spectrometer as a spectrally dispersive element that splits light into its constituent spectral bands prior to the detection by the sensor array. Two primary scanning patterns of spatial scan HSI systems are: point scan (whiskbroom) (Figure 1B) and line scan (pushbroom) (Figure 1C) HSI systems. Compared with the formers, the snapshot HSI systems can simultaneously obtain spectral and spatial information in one image, eliminating the processes of successive scans and relative displacement between the object and the system (Figure 1D) (Halicek et al., 2019a).

**FIGURE 2:**
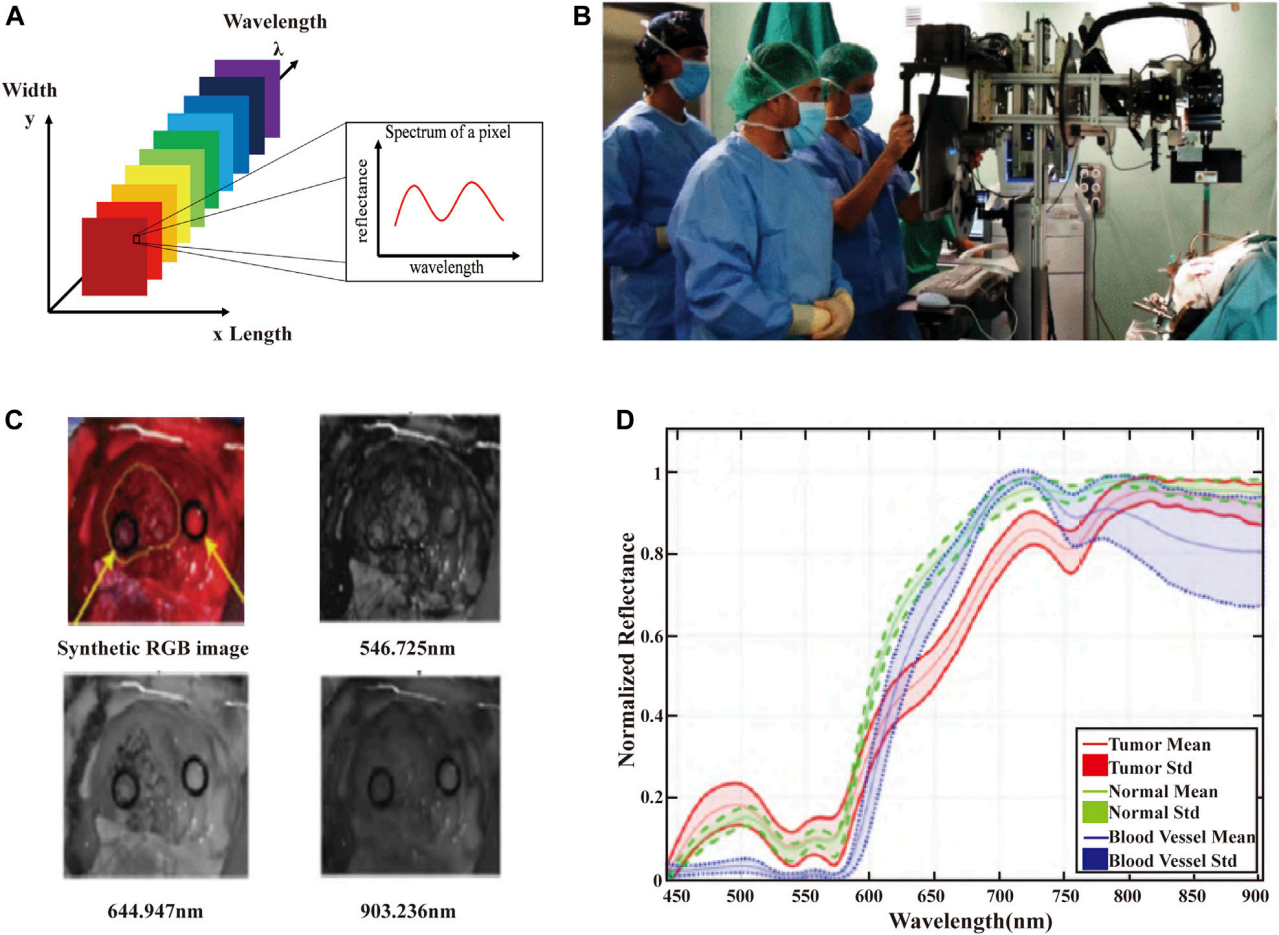
**(A)** The schematic of the HSI data cube. The data measured in the HSI is presented by means of data cubes. Each slice of the data cube includes an image of the scene at a specific wavelength. Each pixel is associated with a spectral response vector, also known as a spectral feature. **(B)** HSI acquisition system used in cerebral diagnosis applications. **(C)** Images at different wavelengths obtained from the brain HSI data cubes. **(D)** Spectral feature information of several brain tumor tissues in the VNIR range at the pixel of ROI (Fabelo et al., 2019a).

HSI systems integrate the advantages of imaging systems and spectroscopic instruments to provide spectral data with spatial resolution. HSI instruments can acquire hundreds or thousands of spectra in an x × y × λ data cube (Figure 2A), where x and y represent the spatial dimensions and *λ* represents the spectral dimension (Sawyer et al., 2017). Figure 2B shows the HSI acquisition system used in cerebral diagnosis. HSI measures the optical properties of brain tissue in broadband electromagnetic wavelengths range. The light interactions (scattering of photons) of brain tissues are captured to generate spectral images of narrow spectral bands (usually 100 or more images). Each image records the relative light absorption or reflectance of one wavelength band, and reveals the biological properties in brain, e.g., chromophores or tissue oxygenation. All these images are assembled into a discrete 3D volume element with two spatial dimensions and one spectral dimension to form a hyperspectral cube (also known as hyperspectral image or hypercube). Figure 2C presents the images at different wavelengths obtained from the brain HSI data cubes. The three-dimensional data cube (hypercubes) are acquired from hyperspectral images that consist of hundreds of images of the same object in different spectral bands. The spectrum of each pixel and image of each slice can present the composition of a specific position, and the spatial and surface feature information (Fabelo et al., 2019a; Manni et al., 2020). Spectral feature information of several brain tumor tissues in the VNIR range at the pixel of regions of interest (ROI) are shown in Figure 2D. The red, green, and blue line represent the spectral characteristics of tumor tissue, normal tissues, and blood vessels.

### 2.2 Hyperspectral Image Processing Methods

The obtained hyperspectral images commonly include many unexpected noise (e.g., brightness non-uniformity, pixel abnormality, redundant regions, data redundancy, etc.) due to the operating conditions of the equipment, research environment, specimen preparation, and other reasons. All these unwanted noises may introduce incorrect and irrelevant signals, affecting the subsequent processing of data. To reduce these variations and extract useful information from hyperspectral images, preprocessing methods are usually used. The workflow of hyperspectral images includes image acquisition, calibration, spectral or spatial pre-processing, downscaling and target-specific detection. Data pre-processing is located at the top of the hyperspectral image processing process and has a direct and important impact on the quality of further analysis. The main purpose of hyperspectral image preprocessing is to compensate for non-uniform illumination and suppress the effects of noisy image elements, extraneous regions and redundant information as much as possible, which can obtain pure images, non-mixed spectral signals and improve the efficiency of subsequent data processing. In the image preprocessing stage, techniques such as image segmentation (ROI selection by masking image regions), noise reduction, image smoothing, flattening, baseline correction, normalization, and image data compression are used. Since hyperspectral images involve both spectral and spatial information, other approaches in the field of spectroscopy analysis and image processing are also suitable for processing hyperspectral data.

More detailed information of the captured scene is obtained in the large amount of data. However, a large increase of the computing power is required to decouple the data with redundant information (Ghamisi et al., 2017). Therefore, it is necessary to employ processing algorithms that can reduce the dimensionality of HS data without losing relevant information. This dimensionality reduction process involves transforming data with high-dimensional features into a significant representation of the data in dimensionality reduction (Audebert et al., 2019). There are two primary dimensionality reduction methods: feature extraction and feature selection (Lu, 2014). Feature extraction algorithms can scale, rotate and reduce the original feature space of HS data utilizing transformation matrices. Common feature extraction methods include principal component analysis (PCA), partial least squares regression analysis, Kernel PCA, linear discriminant analysis, independent component analysis and local linear embedding, all of which retain the information required in practical application (Lv and Wang, 2020). Moreover, it is necessary to choose the most discriminative bands in order to reduce the dimensionality of the data. Optimization algorithms is the most common feature selection algorithms, including genetic algorithm (Mirjalili, 2019), particle swarm optimization (Liu et al., 2021) and ant colony optimization (Sharma and Buddhiraju, 2018).

### 2.3 Hyperspectral Image Analysis Methods

The hyperspectral data cube comprises of a wealth of diagnostic information obtained at the tissue, cellular and molecular levels. All spectral and spatial information in the hyperspectral data cube have important implications for disease screening, clinical diagnosis, and subsequent therapies. The hyperspectral datasets employ advanced image classification technologies to extract, decompose and classify entire spectral information from the acquired data. The purpose of the approach is to associate these molecular features with established disease states by resolving a mixture of spectral and spatial information into intrinsic molecular components expressions. The main classification methods used for hyperspectral imaging are supervised learning and unsupervised learning (Nathan et al., 2018). The classification methods (supervised learning) can be divided into traditional and deep learning methods. Typical methods include Support vector machines (SVMs) (Yi et al., 2014; Manni et al., 2019), Random Forest (Laffers et al., 2016), and K-nearest-neighbor (KNN) (Lu et al., 2016). SVMs is a powerful deep learning technique, the most prominently applied in hyperspectral image data classification and relying on statistical learning theory that separates the linearly separable feature space with maximum margin into classes. In cerebral HSI, SVMs have been already used to identify and classify different types of brain cancers (Fabelo et al., 2018a). SVMs provide good performance for classification of such data when the available number of training samples is limited.

More recently, most tumor recognition models based on HSI technology employ traditional machine learning algorithms. The performance of these tumor detection approaches depends on manually extracting features that are time-consuming, strenuous, and easily influenced by subjective factors. With the rise of deep learning technologies driven by factors such as data mining algorithm and high-performance computing, research on HS images analysis and classification based on deep learning is underway. Using deep learning models can realize autonomous learning of deep, abstract and semantic information from various data. Deep learning utilizes computational models to learn multi-level representations of data through simple combinations rather than non-linear modules, each of which translates the same level of information into a better, more abstract transformation. More deep learning concepts that are relevant and applicable to medical HS images analysis are reviewed in Ref. (Khan et al., 2021). The emergence of deep learning has given rise to more advanced feature extraction techniques by combining spatial and spectral information. Especially in recent years, HSI has begun to achieve promising results in brain disease diagnosis by utilizing cutting-edge deep learning algorithms, such as artificial neural networks (ANNs) (Jolivot et al., 2011), Spectral information dispersion (Guan et al., 2012), and Spectral angle mapping (SAM) (Martin et al., 2012).

Numerous traditional ANN-based algorithms have been used to solve classification and regression problems in the field of biomedical applications. Nevertheless, they perform poorly on independent test data because of overfitting for the plenty of parameters available in HS images. Convolutional Neural Networks (CNN), a very prevalent deep learning algorithm for classifying input images to distinguish objects by identifying patterns and features, has emerged as an effective technique of deep learning for image analysis assignments. They are currently the preferred method for image classification because they exploit spatial features efficiently by executing local patterns in HSI images. Additionally, CNNs are able to capture the correlations between the spectra of a given pixel, exploiting its robustness to training sample variance and to extract features from a large amount of training data, showing great performance in image classification (Halicek et al., 2019b; Halicek et al., 2019c).

In hyperspectral imaging of cerebral experiments, Leave-One-Patient-Out Cross-Validation (LOPOCV) was employed to avoid the double usage of the same patient and three metrics are analyzed, such as the overall accuracy, sensitivity and specificity. Mathematically, sensitivity and specificity are defined as follows:

$$Accuracy=\frac{TP+TN}{\mathrm{TP}+\mathrm{TN}+\mathrm{FP}+\mathrm{FN}}$$ (1)

$$Sensitivity=\frac{TP}{TP+FN}$$ (2)

$$Specificity=\frac{TN}{TN+FP}$$ (3)

Where TP is the number of true positives, FN is the number of false negatives, TN is the number of true negatives and FP is the number of false positives. In addition, the receiver operating characteristic (ROC) curve was calculated and the area under the curve (AUC) metric was provided for each class in the results.

These algorithms inevitably face two main challenges when applied to brain HSI data: limited number of samples and high dimensionality. It is not necessary in other areas of application, but is more prevalent in cerebral HSI due to the variations of spectral features between patients.

## 3 Hyperspectral Imaging in Cerebral Diagnosis

**FIGURE 3:**
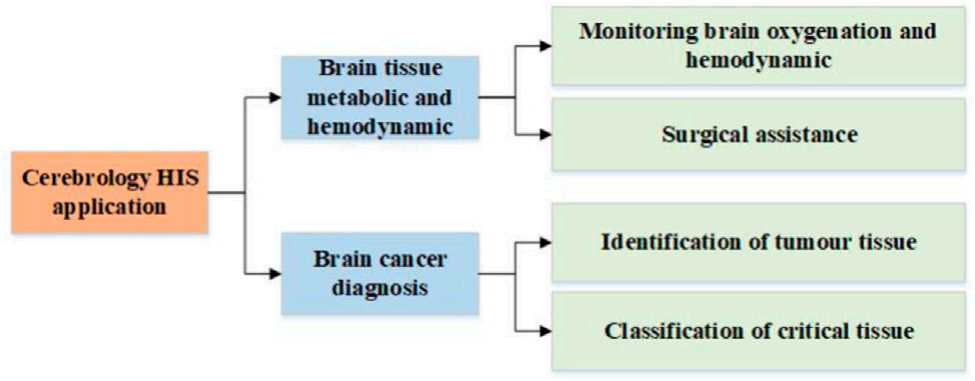
Taxonomy of the current cerebrology HSI applications.

HSI is an emerging technology, of which the application in the biomedical field is still in its early stages. Therefore, there are limited publications on the application of HSI technique in cerebrology. This section summarizes several advanced researches works in this field, which are categorized according to the taxonomy shown in Figure 3. This taxonomy classifies cerebral HSI applications according to the target of application.

### 3.1 Brain Tissue Metabolic and Hemodynamic

Recent biomedical applications of HSI have demonstrated the enormous potential to retrieve *in vivo* hemodynamic and metabolic signals from animal or human brains. HSI provides real-time quantitative information on the biological processes in healthy or diseased brain tissues. This is achieved by measuring the intensity change at multiple wavelengths after reflection, emission, or fluorescence interacts with tissue (Jacques, 2013), which reflecting the changes in optical properties (mainly scattering and absorption) of the target tissue in the brain, and indicating the changes in the biological structure of its constituents and changes in the concentration of chromophores that inherently absorb or emit light (Wang, 2012). In this section, we mainly focus on the research advances of HSI in brain tissue metabolic and hemodynamic.

#### 3.1.1 Monitoring Brain Oxygenation and Hemodynamic

Functional metabolic activity of the human brain is inextricably linked to all biochemical processes associated with normal brain function (Cunnane et al., 2011). Studies have shown a strong relationship between the oxygenation of human brain tissue and its metabolic activation (Malonek and Grinvald, 1996; Devor et al., 2003). Near-infrared HSI is one of the major methods that have been used to achieve brain oxygenation and hemodynamics monitoring (Nguyen et al., 2019). Nosrati, et al. employed near-infrared HSI to measure the concentration changes of oxyhemoglobin (HBO_2_) and deoxyhaemoglobin (HHb), along with the oxidation state of mitochondrial cytochrome c-oxidase (CCO) in the prefrontal cortex on the left and right sides of the human brain simultaneously, which aimed to study the brain metabolic activity of 16 healthy right-handed participants during the simulated driving (aged between 22 and 32) (Nosrati et al., 2016). The results present the absorption spectra of four different tissue chromophores. This data presents the spectral characteristics of HBO_2_, HHb, CCO, and H_2_O in the spectral range 715–900 nm. It is observed that the absorption maxima of HHb and HBO_2_ are located around 760 and 870 nm, respectively. Calculation of concentration changes based on measured spectral features can be applied to the assessment of cerebral oxygen metabolism related events. Giannoni, et al. using Monte Carlo (MC) simulations to demonstrate the applicability of HSI for mapping and quantifying changes in HBO_2_, HHb, and oxCCO concentrations in exposed cerebral cortex under hypoxia (Giannoni et al., 2020). Soon after, the same research group firstly present a novel HSI system for imaging and monitoring of HbO_2_, HHb and CCO in exposed mouse cerebral cortex under different oxygenation state environments (hyperoxia, hypoxia and anoxia) (Giannoni et al., 2021). In this work, the same 8 bands of NIR spectral and 3 bands of visible wavelengths are combined to enhance the image contrast of hemoglobin and oxCCO. Modification and optimization of the HSI system to increase the temporal resolution to the sub-second level enables a more in-depth study of brain function, including brain function activation and neurovascular coupling, etc.

Detecting and measuring brain metabolic signals by imaging allows the identification and localization of changes in brain activity or function under several circumstances, for example, the resting state, through functional activation or stimuli, abnormal physiological environments (e.g., tissue deficiencies oxygen, hyperoxia, and even acute ischemia). These conditions interrupt regular metabolism in a noxious way (e.g., hypoxia and ischemia), and cause damages to brain tissues. Targeting the exposed small animal cortex using HSI is also a preferred option in several studies that evaluating hemodynamic responses and brain tissue oxygenation during induction of hypoxia and hyperoxia (Shonat et al., 1997; Yin et al., 2013; Nishidate et al., 2017; Fu et al., 2020). Shonat, et al. were the first to use HSI to study hemodynamic changes in exposed mouse cortex (Shonat et al., 1997). Mori, et al. reported a study on assessing cortical hemodynamics in rats and humans using HSI system (Mori et al., 2014). In this study, the authors capture continuous spectral data (HS data) of the brain cortical surface in the visible wavelength band and convert these data into optical intrinsic signals to show the measure results of brain surface oxygen saturation. Then, based on previous research, Iwaki et al. successfully demonstrated a new method for predicting cerebral hyperperfusion syndrome (CHS) after bypass surgery for moyamoya (MMD) disease using a hyperspectral imaging system (Iwaki et al., 2021). The authors performed hyperspectral imaging of the cerebral cortex before and after anastomosis in 29 patients with MMD who underwent superficial temporal artery (STA)-middle cerebral artery (MCA) after surgery, and analyzed the changes in oxygen saturation after anastomosis to evaluate its correlation with CHS. Compared with non-CHS patients, CHS patients had significantly higher cerebral cortical oxygen saturation (SO_2_) after anastomosis (33 ± 28 vs. 8 ± 14%, *p* < 0.0001). Therefore, HSI may help pre-identify patients at high risk for postoperative CHS before the onset. This early prediction facilitates early intervention to prevent or reduce irreversible brain disease caused by CHS.

#### 3.1.2 Surgical Assistance

The brain tissue hemodynamics imaging *via* HSI during neural activation is not limited to small animals. Sorts of attempts applied hyperspectral methods to the imaging of exposed human cortex (Klaessens et al., 2011). Currently, many research groups have been devoted to the development of HSI based tools to visualize the hemodynamics during surgeries. Pichette et al. proposed an optical imaging system using snapshot hyperspectral to visualize the hemodynamic behaviours of the brain (Pichette et al., 2016). Firstly, the method has been validated in simulated tissue models to quantify the relative concentrations of up to three absorbing dyes in a mixture with guaranteed accuracy of <10%. Subsequently, the authors applied the tissue model to practical disease treatment with a volunteer of 35-year-old female patient who underwent epileptogenic tissue resection. The HS images were obtained *in vivo* using a hyperspectral HSI Snapshot imaging system covering the spectral range from 481 to 632 nm. This work shows the intra-operative concentration changes of oxyhemoglobin (HbO), deoxyhemoglobin (HbR), and total hemoglobin (HbT)for a single time frame. A reconstructed RGB image for the surface of the brain, realizing visualization of the blood vessels. It also presents the intraoperative concentration changes of HbO, HbR and HbT in a single time period. The relative concentrations in the red region increase while the relative concentrations in the blue region decrease. The results demonstrated that the relative changes in HbO, HbR, and HbT may be aroused through the combined effect of vasomotion, Meyer waves, and response to epileptic spikes.

Several studies monitored brain activities by quantifying changes in oxygen-hemoglobin (ΔC_*HbO2*_), deoxygenated hemoglobin (ΔC_*Hb*_) and oxidative state of cytochrome-c-oxidase (ΔC_*oxCCO*_) concentrations in the cerebral cortex (Bale et al., 2016; Caredda et al., 2020a; Caredda et al., 2020b). Caredda, et al. proposed a method to identify the optimal spectral bands using commercial cameras and assessed brain functional areas and cellular metabolic energy by quantitatively modelling and measuring changes in ΔC_*HbO2*_, ΔC_*Hb*_, and ΔC_*oxCCO*_ through video acquisition during neurosurgery (Caredda et al., 2020a; Caredda et al., 2020b).

This section aims to provide a review of the literatures on HSI based cerebral tissue hemodynamics and metabolism research, which has an important implication for a deeper and broader understanding of the physiologies of brain tissues, to precisely record and map brain activities following neuronal activations.

### 3.2 Brain Cancer Diagnosis

HSI has been proved to be useful in brain disease detection and diagnosis, especially brain cancer. Brain tumors can be classified in terms of their histological and molecular specificity parameters (Louis et al., 2016). Malignant gliomas have become the predominant form of brain tumors in adults, responsible for 2–3% of cancer deaths all over the world (Villa et al., 2018). In addition to methods such as radiotherapy and chemotherapy, surgery has become the best option for treating brain tumors (Kim et al., 2019). However, as brain tumors permeate and spread to the surrounding normal brain tissue, it is difficult for the surgeon’s naked eye to accurately identify tumor tissue from normal brain tissue. Meanwhile, many studies have illustrated the residual tumor tissue to be the main reason for morbidity and mortality during surgeries. Therefore, as a label-free and contactless imaging modality, HSI is a potential tool for tissue boundary identification and classification in brain cancer surgery.

#### 3.2.1 Identification of Tumour Tissue

It is extremely important to identify tumor borders and tumor infiltration into normal brain tissue in order to prevent the removal of excessive normal brain tissue and incomplete resection of residual tumor tissue during neurosurgery. The European project HELICoiD was launched to use HSI technology to identify normal and abnormal brain tissue during neurosurgery (Szolna et al., 2016; Salvador et al., 2017). Fabelo, et al. presented the first image database of *in vivo* HS human brain generated in the HELICoiD (Hyperspectral Imaging Cancer Detection) project (; Fabelo et al., 2019a; Fabelo et al., 2019b; Fabelo et al., 2019c). Ortega, et al. used HSI data to automatically detect and identify pathological sections of human brain tumor tissue taken from 10 different patients with confirmed high-grade glioma (Ortega et al., 2018). The authors employed a custom-built microscopic HS acquisition system that enables clear acquisition of HS images of pathological sections in the VNIR range (400–1000 nm). Thirty-six HS cubes were obtained from these collected pathological section samples, and over 665,000 spectral characteristics of tumor tissue and normal brain tissue from human were labeled. Nevertheless, the large amount of HSI data often contains redundant or irrelevant information. In order to improve the prediction accuracy and reduce the execution time of the classification algorithm, it is central to confirm the most relevant wavelengths for a specific application. Martinez, et al. proposed an optimization algorithm based method to identify the relevant wavelengths through obtaining the correlation spectra of the visible and near-infrared regions to establish the SVM model (Martinez et al., 2019). In this study, the *in vivo* human brain cancer database was derived from 26 HS images of 6 adult patients. To determine the minimum number of sampling wavelengths in HS images, the authors evaluated different band selection algorithms using a supervised classifier. Using the 48 selected bands gives better quantitative and qualitative results than using the full band in some cases.

Processing hyperspectral images *in vivo* is difficult for the high-dimensional nature of the HS data, of which the real-time processing is a challenge. Ravì, et al. introduced a new dimensionality reduction scheme (named Fixed Reference T-distributed Stochastic Neighbours, FR-t-SNE) and a novel processing pipeline to acquire detailed tumor classification maps for margin determination in brain surgery (Ravi et al., 2017). In this work, an extension of the FR-t-SNE method was used to decrease the data dimensionality of the HS database and the embedded results were semantically segmented using the Semantic Text Forest (STF) method to achieve brain tumor tissue classification.

Deep learning-based HSI data analysis showed a broad prospect in the field of intelligent assisted diagnosis. Fabelo, et al. proposed a deep learning-based framework to identify human brain tumors, and achieved an overall accuracy of 80% using multi-class classification on a dataset of 26 *in vivo* HSI samples (Fabelo et al., 2019a). Manni, et al. employed a 3D-2D hybrid convolutional neural network for the extraction of spectral-spatial features to classify brain tissues. Based on this network, tumor, normal tissue and blood vessels in the human brain were effectively distinguished. Compared to traditional feature extraction methods, the deep learning-based methods transform informative features from raw images utilizing hierarchical structures. The 3D–2D hybrid CNN achieves the best results with a mean accuracy of 80%, sensitivity of 76, 68, 74, 96%, specificity of 87, 98, 92, 87%, and AUC of 78, 70, 84, 91%, for normal, tumor, blood vessels and background, respectively (Manni et al., 2020). Furthermore, Hao, et al. reported a multiple deep model fusion (include three neural networks) based extraction method to achieve an overall accuracy of 96.34% for the identification of GBM tumors (Hao et al., 2021). This method employed 1-D deep neural network (1D-DNN) and 2-D convolution neural network (2D-CNN) to extract spectral characteristics and spectral spatial characteristic for the HSI classification of human brain. The authors also utilized edge-preserving filters to fuse and optimize spectral and spectral-spatial classification results and used fully convolutional network (FCN) to segment the background from images. The experimental data showed that the method has good classification performance.

#### 3.2.2 Classification of Critical Tissue

It is also important to classify the critical parts of the brain tissue in real time during neurosurgery or experiments. Fu, et al. provided a method using HSI to demonstrate the possibility of differentiate between infarcted and normal brain tissue (Fu et al., 2020). The authors adopted the specific value of spectral reflectance at 545 and 560 nm (R545/R560) to recognize the spectral features of normal rat tissues and tissues with different levels of ischemia, which evaluated the utility value of the rat ischemic stroke model. The results illustrated that hyperspectral images processed with the ratio of R545 and R560 could not only recognize early cerebral ischemia within 1 h, but also accurately display ischemic regions. Fabelo, et al. used a semi-automatic approach based on a SAM algorithm to define 4 distinct classes, which included normal tissue, tumor tissue, blood vessels, and background (Fabelo et al., 2019b). Then, Urbanos, et al. used three different algorithms to classify brain tissue *in vivo* from 13 patients with high-grade gliomas, including support vector machines (SVM), random forests (RF), and convolutional neural networks (Urbanos et al., 2021). In this work, the authors evaluated three classification algorithms with an HSI snapshot camera with limited wavelength bands and distinguished five different brain tissue types (tumor, arterial blood vessel, venous blood vessel, dura mater and healthy tissue). According to the different training conditions, the overall accuracy results obtained from the experiments ranged from 60 to 95%.

In order to facilitate the operation of the surgeon during tumor resection, it is essential to develop a real-time classification system that can delineate the boundaries of the cancer with high accuracy. Fabelo, et al. proposed a novel algorithm of brain cancer detection to help neurosurgeons to classify brain tumor tissues, which consisted of a hybrid framework that combined both supervised and unsupervised machine learning methods (Fabelo et al., 2018b). The authors interfaced the calculator with a hardware gas pedal, allowing the system to provide neurosurgeons with a categorized map of scenes obtained in approximately 1 min during surgery. These preliminary results extracted from the supervised classification of pre-labeled tissues by experts showed that normal tissue, tumor tissue, blood vessels and background could be accurately distinguished with an overall accuracy higher than 99%. Due to the high-dimensional nature of the data acquired with the HS camera and the need to perform intraoperative surgical guidance tools in real time, a highly parallel high-performance processing platform must be used to process the HS data. Among the various available parallel technologies, the Graphics Processing Unit (GPU) is the most attractive solution, which can execute complex, intrinsically parallel algorithms on large amounts of data. Florimbi, et al. employed GPU technology to classify the largest (worst-case) image in the database in less than 3 s, which satisfied the surgery limitation of real-time setting within 1 min, and became a potential method for hyperspectral video processing in the immediate future (Florimbi et al., 2020).

Although machine learning methods and deep learning method are used extensively in brain cancer diagnosis, the integrity of data information and the timeliness of data processing are still the shortcomings of these methods (Barberio et al., 2021). More recently, several research groups are focusing on hybrid deep learning method for clinical diagnosis of brain cancer (Rinesh et al., 2022).

### 3.3 Hyperspectral Imaging Application Summary

To sum up, we summarized the most relevant works in the field of cerebral diagnosis using HSI in Table 1. This table is organized as follows: 1) the type of application; 2) the type of subjects involved in each study; 3) the type of sample involved in each study; 4) the spectral range of HSI technology; 5) the data processing and analysis methods or algorithms employed.

**TABLE.1:** Summary of HSI applications in cerebral diagnosis.

Application	Year	Study Subjects	Type of the Sample	Spectral range (nm)	Data processing and analysis methods/algorithms	References
Monitoring brain oxygenation and hemodynamic	2015	Animal/Rats	*in-vivo*	484–652	MBLL	Konecky et al. (Konecky et al., 2015)
	2016	Human/Brain	*in-vivo*	700–900	ICA	Nosrati et al. (Nosrati et al., 2016)
	2018	Animal/Mice	*in-vivo*	450–998	MBLL	Giannoni et al. (Giannoni et al., 2018)
	2019	Human/Brain	*in-vivo*	650–1100	LSM	Nguyen et al. (Nguyen et al., 2019)
	2019	Animal/Rats	*in-vivo*/*ex-vivo*	400–720	-	Fu et al. (Fu et al., 2020)
	2020	Animal/Mouse	*in-vivo*	780–900	MC	Giannoni et al. (Giannoni et al., 2020)
	2021	Animal/Mice	*in-vivo*	780–900	MC	Giannoni et al. (Giannoni et al., 2021)
	2021	Human/Brain	*in-vivo*	400–800	-	Iwaki et al. (Iwaki et al., 2021)
Surgical assistance	2014	Animal/Rats Human/Brain	*in-vivo*	400–800	LSM	Mori et al. (Mori et al., 2014)
	2016	Human/Brain	*in-vivo*	481–632	LSM	Pichette et al. (Pichette et al., 2016)
	2020	Human/Brain	*in-vivo*	675–1000	MBLL	Caredda et al. (Caredda et al., 2020a)
	2020	Human/Brain	*in-vivo*	-		Caredda et al. (Caredda et al., 2020b)
Identification of tumor tissue	2017	Human/Brain	*in-vivo*	600–720		Bravo et al. (Bravo et al., 2017)
	2017	Human/Brain	*in-vivo*	400–1700	DNN, FR-t-SNE, STF	Ravi et al. (Ravi et al., 2017)
	2018	Human/Brain	*ex-vivo*	400–1000	SVM, ANN, RF	Ortega et al. (Ortega et al., 2018)
	2018	Human/Brain	*in-vivo*	-	PCA, SVM, k-means, KNN	Torti et al. (Torti et al., 2018)
	2019	Human/Brain	*in-vivo*	400–1000	SVM, k-means, GA, ACO, PSO	Martinez et al. (Martinez et al., 2019)
	2019	Human/Brain	*in-vivo*	400–1000	PCA, KNN, SVM, SAM, CNN	Fabelo et al. (Fabelo et al., 2019a)
	2020	Human/Brain	*ex-vivo*	400–1000	CNN	Ortega et al. (Ortega et al., 2020)
	2020	Human/Brain	*in-vivo*	400–1000	SVM, CNN	Manni et al. (Manni et al., 2020)
	2021	Human/Brain	*in-vivo*	400–1000	PCA, CNN, DNN, FCN	Hao et al. (Hao et al., 2021)
Classification of critical tissue	2018	Human/Brain	*in-vivo*	400–1000	KNN,SVM, PCA	Florimb et al. (Florimbi et al., 2018)
	2018	Human/Brain	*in-vivo*	400–1000	SVM, KNN, SAM, FR-t-SNE	Fabelo et al. (Fabelo et al., 2018a)
	2018	Human/Brain	*in-vivo*	400–1700	SVM, PCA, KNN	Fabelo et al. (Fabelo et al., 2018b)
	2019	Human/Brain	*in-vivo*	400–1000	PCA, KNN, SVM, SAM, CNN, DNN	Fabelo et al. (Fabelo et al., 2019d)
	2020	Human/Brain	*in-vivo*	400–1000	PCA, KNN, k-means, SVM, SAM	Florimb et al. (Florimbi et al., 2020)
	2021	Human/Brain	*in-vivo*	400–1000	SVM,EMD	Baig et al. (Baig et al., 2021)
	2021	Human/Brain	*in-vivo*	-	BLU, SVM, SAM, CNN, DNN	Cruz-Guerrero et al. (Cruz-Guerrero et al., 2020)
	2021	Human/Brain	*in-vivo*	655–975	SVM, RF, CNN	Urbanos et al. (Urbanos et al., 2021)
	2022	Human/Brain	-	400–1300	MFNN, SVM, K-Means	Rinesh et al. (Rinesh et al., 2022)

Data analysis methods/algorithms: MBLL, modified Beer-Lambert law; ICA, independent component analysis; LSM, least square method; SAM, spectral angle mapper; MC, monte carlo framework; SVM, support vector machines; PCA, principal component analysis; RF, random forest; ANNs, Artificial Neural Networks; FR-t-SNE, fixed reference t-distributed Stochastic Neighbours; STF, semantic texton forest; MFNN, multilayer feed forward Neural Network; CNN, convolutional neural network; FCN, fully convolutional network; BLU, blind linear unmixing; KNN, k-nearest neighbors; EMD, empirical mode decomposition; DNN, deep neural network; MFNN, multilayer feed forward Neural Network.

## 4 Discussion and Outlook

Based upon the above studies, it has been demonstrated that hyperspectral imaging has great potential for applications in cerebral diagnosis, for the excellent sensitive of the spectral signal to the biophysical and biochemical characteristics of cell and tissue samples.

Compared with traditional imaging methods, the outstanding advantage of HSI lies in the simultaneous generation of tissue structure information and hemodynamic parameters, which cannot be achieved by conventional technologies (e.g., CT or MRI). Furthermore, HSI is a real-time online, non-invasive, non-ionizing imaging technique that solves the sampling and time-consuming issues associated with hematoxylin and eosin (H&E) and 2,3,4-triphenyltetrazolium chloride (TTC) staining. Moreover, compared to common RGB images, HS images have more spectral channels and higher spectral resolution, which may contain more useful information about the surgeon’s tissue physiology and pathophysiolog. In brain tissue oxygenation and hemodynamics, HSI techniques have superior spectral resolution in imaging light-absorbing chromophores, for example, HbO_2_, HHb and oxCCO. Although small animals remain a prime target for metabolic activity monitoring, the study of humans in neurosurgery is becoming more common and can provide important insights into the effects of different stimuli on the human brain.

In summary, these related literatures present promising results for many kinds of cerebral diagnosis applications according to biomedical HSI and for surgical guidance. However, there are still some limitations in technology insufficiency and small datasets. Pilot studies encouraged further research of all organ systems to determine the role of HSI in clinical. For the advantages of contactless, non-invasive, label-free, and non-ionizing, HSI is a promising imaging method with great potential. However, the reliability, reproducibility, and generalizability should be further validated before the extensive usage in biomedical applications.

Although many studies have demonstrated its potential in cerebral diseases, these areas still suffer from variability in hardware and software, short of comparison or unified standard evaluation system, and lack of sufficiently large sample sizes. We can improve from the following aspects: 1) The limitation of imaging depth. Combining other techniques such as photoacoustic imaging systems to improve imaging depth remains to be studied (Liu et al., 2020b). 2) Performance improvement of HSI systems. In fact, the available hyperspectral imaging systems are still relatively bulky, and are not suitable for minimally invasive surgery. The continuous improvement of HSI systems in imaging, spatial resolution, spectral range, and resolution will enable portable and smaller HSI devices, and facilitate widespread medical applications in cerebral diagnosis. In addition, scholars have also begun to use multiple HS cameras to acquire more images in the broad spectral band, with the aim of analyzing different characteristics of different types of normal brain and tumor tissue *in vivo* (Leon et al., 2021a, Leon et al., 2021b). 3) Real-time processing of HSI video. HSI techniques are combined with deep learning processes to establish models for clinical diagnosis and surgical treatment of brain disorders. However, processing the huge amount of data captured by HSI sensors and improving the efficiency of data processing are the main issues for realizing real-time HSI video processing in the future (Sancho et al., 2021). 4) Formation of a multimodal imaging system. Fluorescence spectral imaging and Raman spectral imaging methods also have been introduced by researchers for different kinds of biological imaging (Sun et al., 2021; Wang et al., 2021). Furthermore, hyperspectral imaging combined with other biomedical imaging modalities to form a multimodal imaging system which can completely explain and predict the behavior of brain tissue or brain cells. 5) Establishment unified diagnostic standard. By establishing a database of characteristic spectra of various parts of the brain tissue and different brain diseases, online sharing is realized, and a unified diagnostic standard is continuously formed. Finally, this review systematically revealed the application of HSI method on the exposed cortex in cerebral surgery. However, the HSI Technology covers also the diffuse imaging of the brain with wide applications, including neonatal, aging, neurodegenerative, and cardiac arrest/surgery brain monitoring, which would not be discussed here. In conclusion, biomedical HSI remains an explosively expanding field, and new-type technological advances may lead to new discoveries and enhanced understanding of the mechanisms underlying brain function.
